# Genotoxic stress signalling as a driver of macrophage diversity

**DOI:** 10.15698/cst2022.03.265

**Published:** 2022-02-14

**Authors:** Ana Kasapi, Antigoni Triantafyllopoulou

**Affiliations:** 1 Department of Rheumatology and Clinical Immunology and Institute of Microbiology, Charité University Medical Center, D-10117 Berlin, Germany.; 2 German Rheumatism Research Center, a Leibniz Institute, D-10117 Berlin, Germany.

**Keywords:** DNA damage, ATR, ATM, innate immunity, macrophage programs, chronic inflammation

## Abstract

Tissue macrophages arise from yolk sac, fetal liver and hematopoietic progenitors and adopt diverse transcriptional programs and phenotypes, instructed by their microenvironment. In chronic inflammation, such as in chronic infections, autoimmunity, or cancer, tissue microenvironments change dramatically thus imprinting new programs on tissue macrophages. While stress is a known driver of carcinogenesis in epithelial cells, emerging evidence suggests that macrophage responses to genotoxic stress are embedded in their ‘physiologic' immune and tissue healing programs and in most cases do not lead to myeloid malignancies. The role of genotoxic stress as an instructor of macrophage-mediated immune defense and tissue remodeling is only beginning to be understood. Here, we review the evidence showing that genotoxic stress, which macrophages and their precursors face upon encountering inflammatory and/or growth signals, instructs their transcriptional programs, by activating non-canonical, cell-type specific DNA Damage Response (DDR)-driven signaling pathways. We propose that immune-cell specific, DDR-instructed programs are crucial for tissue homeostasis as well as for the maintenance and resolution of inflammatory responses in infection, cancer, autoinflammatory and autoimmune microenvironments.

## INTRODUCTION

Macrophages are innate immune cells that play an essential role in host defense, maintenance of homeostasis and tissue repair. They phagocytose apoptotic cells, cellular debris or foreign material, sense and respond to microenvironmental signals using scavenger, pattern recognition, nuclear hormone and cytokine receptors [1, 2] promoting homeostasis and immune defense. Tissue resident macrophages originate from the yolk sack and fetal liver and populate tissues before birth. Subsequently, they proliferate locally and self-renew during adult life [3–5]. Their phenotypes are highly diverse and depend on the organ that they populate and their microenvironmental niches. Thus, Kupffer cells in the liver, microglia in the brain, Langerhans cells in the skin, red pulp macrophages in the spleen, or alveolar macrophages in the lung possess distinct phenotypic features, mirroring their diverse transcriptional and epigenetic imprinting [6–8]. Macrophage diversity is further reflected by distinct activation states, out of which, canonical M1, arising after *in vitro* stimulation with lipopolysaccharide (LPS) and interferon (IFN)-γ and having a pro-inflammatory function and alternative M2, generated after *in vitro* stimulation with interleukin (IL)-4 and promoting tissue healing, represent the extremes of a broad, continuous spectrum [9].

Macrophages, like epithelial cells, face genotoxic stress and in response activate an evolutionary conserved signaling cascade, the DNA Damage Response (DDR). The canonical functions of the DDR, common across cell types and species, promote DNA repair and preserve genome integrity. In pre-cancerous epithelial cells, the DDR may act as a barrier to tumor progression. Accordingly, oncogene activation in preneoplastic and neoplastic lesions leads to replication stress and increased senescence. DDR inhibition suppresses senescence, while ectopic activation of one of the main DDR kinases is sufficient for inducing senescence. Furthermore, both DDR activation and senescence induction decrease as lesions progress to carcinomas [10–12]. Interestingly, in patients of ulcerative colitis, a subtype of inflammatory bowel disease with a higher risk of colon cancer, increased senescence correlated with macrophage infiltration and was partly dependent on their release of nitric oxide (NO) [13]. Although this study did not address the direct relevance of components of the DDR machinery in the process, it did show that NO may activate the DDR *in vitro*. Thus, the DDR in pre-cancerous settings introduces a barrier to carcinogenesis and is potentially activated by NO-producing macrophages.

**Figure 1:**
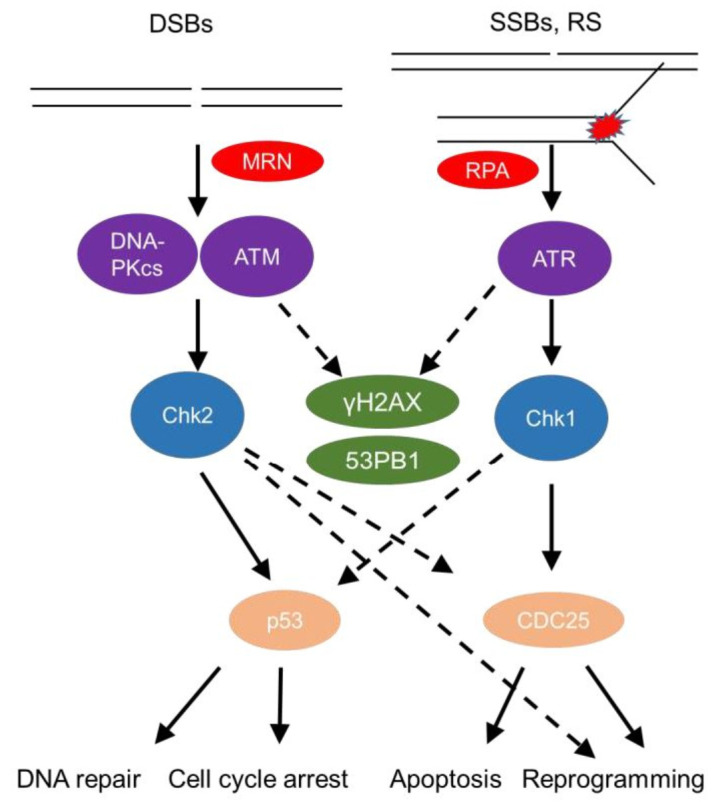
FIGURE 1: The DNA Damage Response. There are two major arms to the DDR, one designed to deal with double strand breaks (DSBs), which are sensed by the MRN (Mre11, Rad50 and Nbs1) complex, alerting the DNA-PKcs (DNA-dependent protein kinase, catalytic subunit) and ATM (ataxia-telangiectasia mutated) kinases, which further phosphorylate Chk2 (checkpoint kinase 2), and the other one sensing single strand breaks (SSBs) and replication stress through RPA (Replication protein A), activating the ATR (Ataxia telangiectasia and Rad3 related)-Chk1 (checkpoint kinase 1) axis and both arms phosphorylating γH2AX (phosphorylated H2A histone family member X) and 53BP1 (p53-binding protein 1). Major regulators of cell fate such as p53 and cell cycle regulators (for instance cell division cycle 25 phosphatase (CDC25)) get activated and these events, based on the severity of the damage lead to either DNA repair, cell cycle arrest to allow the cell to repair this damage. If damage is substantial and beyond repair, the cell will either go into senescence (permanent cell cycle arrest) or apoptosis (cell death). Alternatively, these pathways can activate non-canonical programs that drive the cell into re-programming and differentiation.

The DDR senses DNA damage, such as single strand (ss) or double strand (ds) DNA breaks, as well as stalled replication forks (**Figure 1**). This sensing results in either repair of the damaged site or, in cases of substantial damage, cell cycle arrest or cell death [14, 15]. DsDNA breaks activate mainly the phosphatidylinositol 3-kinase (PI3K) - like protein kinases Ataxia telangiectasia mutated (ATM) and DNA-dependent protein kinase (DNA-PK; **Figure 1**). Sensing of dsDNA breaks by the Mre11-Rad50-NBS1 (MRN) Complex, binds and activates the kinase ATM. DNA-PK, a nuclear serine/threonine protein kinase, is a trimeric complex, composed by a catalytic subunit (DNA-PKcs) and two DNA binding subunits, Ku70 and Ku80. The latter sense and bind dsDNA breaks and activate DNA-PKcs kinase activity to initiate DNA repair [16, 17]. Interestingly, autoantibodies against Ku70/Ku80 [18] are found in sera of autoimmune disease patients, including patients with systemic lupus erythematosus and polymyositis-dermatomyositis overlap [19] highlighting the role of the DDR in autoimmune pathologies.

The pathway by which repair of dsDNA breaks occurs relies significantly on whether DNA end resection happens [20]. In the absence of end resection, blunt ends are joined in a non-homologous end joining (NHEJ)-dependent manner. Once nucleases such as Mre11 and CtIP have processed the ends flanking double strand breaks (DSBs), generating sticky ends, three other pathways compete for repairing the break, namely homologous recombination (HR), single strand annealing (SSA), and alternative end joining (Alt-EJ) [20]. Detailed reviews of repair pathways and their outcomes have been given elsewhere [20, 21]. The role of individual repair pathways on macrophage development has not been elucidated.

SsDNA breaks and stalled replication forks coated by the damage sensor molecule Replication protein A (RPA), recruit the kinase ATM- and Rad3-related (ATR) and its partner protein ATR interacting protein (ATRIP) [22]. Subsequently, downstream mediators, checkpoint kinases, Chk1 and Chk2 (when ATM is deficient) [23] are activated and help to amplify the DDR [24], recruit repair factors, or activate regulators of cell fate, such as the master transcription factor p53 (**Figure 1**) [12]. A timely termination of the DDR is executed through dephosphorylation of DDR components by phosphatases, such as Protein phosphatase 2A (PP2A) and wild type p53-induced phosphatase 1 (Wip1), as well as through proteolysis by the ubiquitin-proteasome pathway (UPP) [21].

The interplay of the DDR with the immune response has been extensively reviewed [25]. Apoptotic and senescent cells are recognized and removed by phagocytic cells [26]; ATM activation in non-immune cells upregulates natural killer group 2 member D (NKG2D) ligands, activating natural killer (NK) cells at the site of injury [27]; p53 often induces an inflammatory response in the context of tumorigenesis [28], while also activating antigen-presenting-related molecules in macrophages [29, 30]. While this body of work has provided important insights on how the immune system responds to non-immune cells that have activated the DDR, our knowledge of how immune cell-intrinsic DDR activation modifies immune programs is much more restricted.

Given the significance of preserving genomic integrity for cancer development, the canonical functions of the DDR have been the focus of intense investigation. A proteomic screen demonstrated 15 years ago that the spectrum of DDR signaling proteins is very wide, including protein networks that are not directly linked to cell cycle or cell death pathways, such as proteins in the insulin-IGF1 (insulin-like growth factor)-PI3K- AKT pathways. This raised the possibility that the functional implications of DDR signaling go well beyond its well-characterized role in DNA repair and maintenance of genome integrity [31]. Taking this a step further, an increasing body of literature has uncovered non-canonical, cell-type specific functions of the DDR (reviewed in [32, 33]). Here, we will review non-canonical immune-cell specific functions of the DDR, placing more emphasis on its role as a cell-autonomous regulator of innate immunity and macrophage re-programming.

## RAG RECOMBINASES REGULATE ADAPTIVE AND INNATE LYMPHOCYTE DEVELOPMENT VIA THE DDR

**Figure 2:**
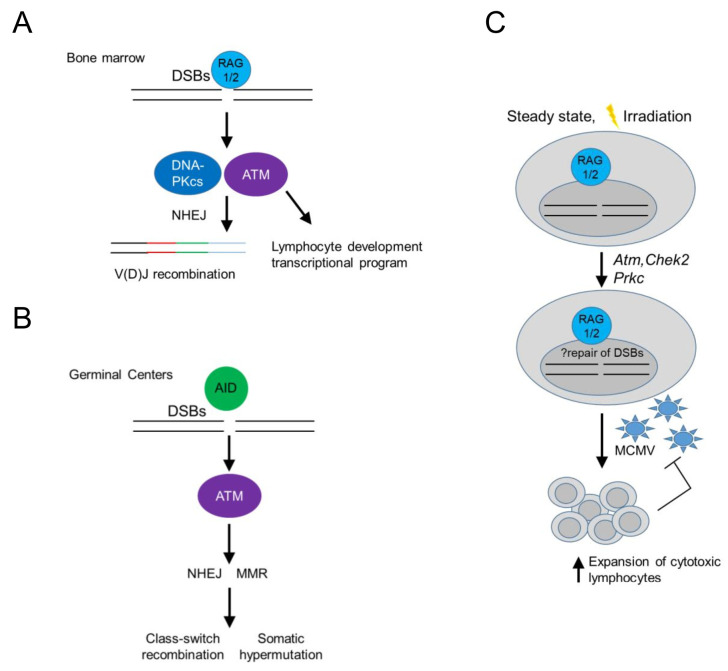
FIGURE 2: RAG introduce programmed dsDNA breaks during the assembly and diversification of lymphocyte antigen receptor gene may promote cellular fitness of cytotoxic lymphocytes. **(A)** RAG (recombination-activating genes)-induced dsDNA breaks activate DNA-PKcs (DNA-dependent protein kinase, catalytic subunit) and ATM (ataxia-telangiectasia mutated), initiating a canonical DDR that leads to double strand break repair by NHEJ (non-homologous end joining), while ATM signaling additionally induces a non-canonical DDR that regulates cell type-specific, developmental programs in B cells in the bone marrow. **(B)** In mature B cells, antigen stimulation and co-stimulatory signals induce AID (activation-induced deaminase) – dependent breaks that activate ATM, initiating a non-canonical DDR that promotes activated B cell receptor diversification in germinal centers, employing several repair pathways, such as mismatch repair (MMR). **(C)** RAG-induced dsDNA breaks upregulate the expression of DDR-related genes, such as *Atm (*encoding ataxia telangextasia mutated)*, Chek2* (encoding checkpoint kinase 2) and *Prkdc* (encoding DNA-dependent protein kinase, catalytic subunit (DNA-PKcs)) in NK and CD8 T cells, which may increase their ability to deal with genomic instability upon virus-induced proliferation and cellular stress, thus facilitating their population expansion and cytotoxic effector functions.

In the context of immune cell development, DDR signaling in developing B cells has served as a paradigm of an immune cell-type specific, DDR-mediated signaling pathway operating at homeostasis. In developing B cells, recombination-activated gene proteins (RAG) introduce programmed dsDNA breaks during the assembly and diversification of lymphocyte antigen receptor genes [32, 34] (**Figure 2**). RAG-induced dsDNA breaks activate DNA-PKcs and ATM, initiating a canonical DDR that leads to double strand break repair by NHEJ, while ATM signaling additionally induces a non-canonical DDR that regulates cell type-specific, developmental programs in B cells [32, 35, 36] (**Figure 2A**). In mature B cells, antigen stimulation and co-stimulatory signals induce activation-induced deaminase (AID) – dependent ssDNA and subsequent dsDNA breaks that activate ATM, initiating a non-canonical DDR that promotes class switch recombination in germinal centers leading to the differentiation of activated B cells into plasma cells [32] (**Figure 2B**).

Interestingly, RAG-activated DDR signaling may also promote cellular fitness of innate or adaptive cytotoxic lymphocytes [37, 38], as NK cells or CD8 T cells from *Rag2* deficient mice failed to expand *in vivo* following murine cytomegalovirus (MCMV) infection and displayed increased levels of phosphorylated histone H2AX (γH2AX) at homeostasis and after irradiation (**Figure 2C**). Rag2 deficient NK cells showed at homeostasis decreased basal expression of *Atm, Chek2* and *Prkdc* (encoding DNA-PKcs) and *Xrcc5* (encoding Ku80), suggesting a diminished ability to repair DNA lesions and thus genomic instability in response to proliferation or cellular stress [38] (**Figure 2C**). The use of RAG-fate map mice revealed that half of bone marrow and a minority of peripheral NK cells previously expressed RAG1 [37, 39] and prior RAG expression correlated with a more immature and less terminally differentiated phenotype [37]. These data left a number of unanswered questions open: 1. Is there is a differential program expressed by NK cells that previously expressed RAG following infection? 2. Were the observed increased levels of γH2AX in RAG deficient NK cells ATR dependent (since ATM and DNA-PKcs were expressed in lower levels)? 3. Do such findings play true only in the context of MCMV infection in the C57BL/6 background in mice or also in different genetic backgrounds, following other sources of cellular stress and in humans? 4. What is the role and mechanism by which RAG may regulate the expression of ATM and DNA-PKcs in NK cells, which are not known to have induced dsDNA breaks during their development and do not undergo V(D)J recombination? 5. Finally, whether long-lived memory NK cells have all expressed RAG during their development is another interesting open question.

## THE MRN COMPEX AND ATM CONTROL STEADY STATE MACROPHAGE RESPONSES TO MCSF

**Figure 3:**
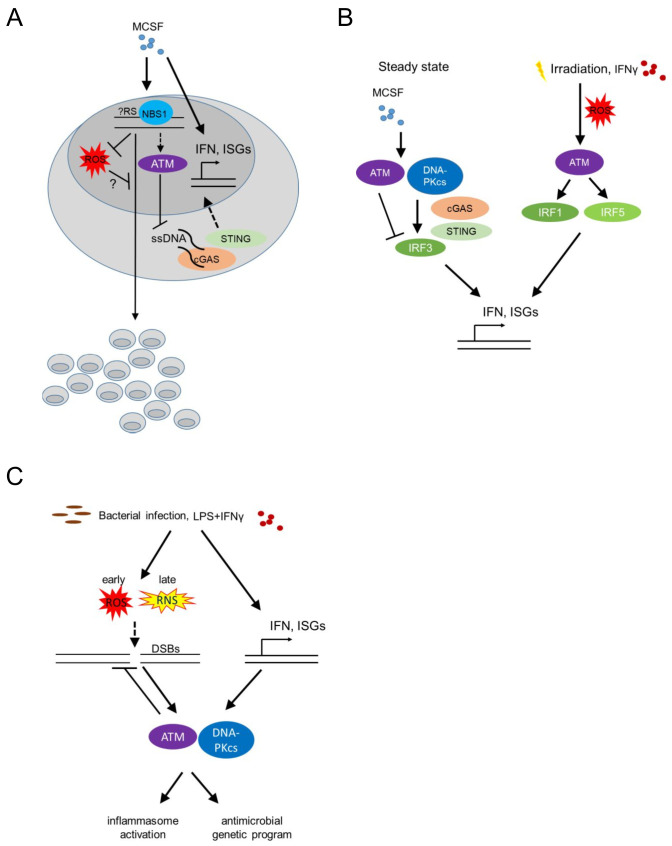
FIGURE 3: ATM regulates macrophage responses to MCSF, LPS and IFN-γ. **(A)** MCSF (macrophage colony stimulating factor) induces macrophage proliferation and differentiation, while activating type I interferon (IFN) responsive genes as well as potentially introducing genotoxic stress sensed by the MRE complex. This in turn downregulates ROS (reactive oxygen species) and activates downstream players of the DDR (such as ATM), thus suppressing genomic instability and promoting population expansion. Activated ATM in turn suppresses ssDNA accumulation in the cytoplasm and thus cGAS (Cyclic GMP-AMP synthase) - STING (Stimulator Of Interferon Response cGAMP Interactor)-induced IFNβ activation and interferon responsive genes. **(B)** At steady state MCSF upregulates type I IFN responsive genes by activating DNA-PK, STING and subsequently IRF3, while ATM itself suppresses the type I IFN response. Upon irradiation, increased expression of type I IFN responsive genes requires ATM-mediated IRF1 activation. Upon irradiation or following stimulation with IFNγ, ROS-induced ATM activation additionally leads to IRF5 upregulation, thus further activating type 1 IFN responses. (IRF-Interferon Regulatory Factor, ISG-Interferon Signature Genes). **(C)** Bacterial infection as well as stimulation with LPS and IFN-γ in BMDMs cause genotoxic stress, through the generation of ROS and reactive nitrogen species (RNS), thus activating ATM and DNA-PKcs in a type I IFN dependent manner. This leads to the upregulation of an antimicrobial macrophage genetic program as well as inflammasome activation.

Bone marrow-derived macrophages (BMDM) respond to stimulation with recombinant Colony Stimulating Factor 1 (CSF1, also known as Macrophage Colony Stimulating Factor, MCSF) by proliferation and differentiation. The latter is accompanied by a significant upregulation of type I IFN responsive genes ([40] and own unpublished data; **Figure 3A**). Proliferation of BMDM is compromised in the absence of phosphopeptide-binding Nijmegen syndrome protein 1 (NBS1), a component of the dsDNA break-sensing MRE complex [41]. BMDM genetically deficient in *Nbs1* displayed increased chromosomal aberrations and increased basal levels of reactive oxygen species (ROS), following growth in MCSF-containing media [41] (**Figure 3A**). Though treatment with MCSF did not induce phosphorylation of the histone H2AX [24] (γH2AX), an early marker of dsDNA break-induced DDR activation, detected as nuclear foci by immunofluorescence, the data suggest that MCSF-induced macrophage precursor proliferation alone imposes a certain level of genotoxic stress that activates the DDR machinery. Thus, DDR activation may become a limiting factor for macrophage population expansion in the context of tissue macrophage self-renewal or proliferation following infection or tissue damage, controlling successful tissue healing and maintenance of homeostasis. Accordingly, bone marrow-derived and tissue resident macrophage proliferation *in situ* in the peritoneal cavity, following zymosan injection intraperitoneally (a model of toll-like receptor (TLR)2-driven peritonitis), an MCSF dependent process [42] was compromised in *Nbs1* deficient mice [41].

Beyond its speculative role in regulating proliferation in response to MCSF, the DDR, and specifically ATM was found to suppress the upregulation of IFN response genes that is induced by MCSF in BMDM at steady state [40, 43]. BMDM from *Atm* deficient mice grown in medium containing MCSF showed increased levels of *Ifnb1* and *Mx1* transcripts. Furthermore, *Atm* deficient mice displayed increased IFN-beta production *in vivo* (measured using a sensitive IFN-beta luciferase reporter mouse model [44] at steady state, as well as after infection with vesicular stomatitis virus (VZV); **Figure 3B**). The same was true after *in vitro* infection of BMDM with *Listeria monocytogenes* or following irradiation or treatment of BMDM with etoposide, establishing a wider role of ATM in regulating BMDM type I IFN responses at homeostasis (where no dsDNA breaks are detectable), infection or classical genotoxic stress induced by dsDNA breaks (*i.e.* irradiation or etoposide). In the absence of ATM, macrophages accumulated ssDNA in the cytoplasm at steady state and activated the cytoplasmic nucleic acid receptor STING (stimulator of interferon genes). The latter was required for the increased interferon beta expression of *Atm* deficient BMDM [43] (**Figure 3B**).

## ATM-REGULATION OF TYPE I IFNs IN MACROPHAGES AT STEADY STATE VS FOLLOWING IRRADIATION

Interestingly, the pathways leading to increased type I IFN expression by BMDM in response to MCSF at steady state versus after irradiation-induced genotoxic stress are dramatically different. DNA-PK, STING, IFN regulatory factor (IRF) 3 and IFN alpha/beta receptor 1 (IFNAR1) were required for type I IFN upregulation by MCSF at steady state, while ATM itself suppressed basal MCSF-dependent type I IFN responses (**Figure 3B**). In contrast, following irradiation, increased type I IFN responsive genes were STING independent, but required ATM-mediated IRF1 activation [40] (**Figure 3B**). The latter finding contradicted prior results [43], potentially because in the initial study the basal increase of type I IFN responsive genes in the absence of STING at steady state masked its effect following irradiation [40, 43]. What activates ATM to suppress MCSF-mediated tonic type I IFNs in BMDM at steady state remains an intriguing question, particularly because at homeostasis, without irradiation, neither dsDNA breaks nor cytoplasmic ssDNA are detectable.

## IRRADIATION-INDUCED ATM SIGNALING IS COUPLED TO PRO-INFLAMMATORY PROGRAMS VIA ROS

Extensive DNA damage and sensing of nucleic acids trigger pro-inflammatory programs (reviewed in [45, 46]). These studies have linked dsDNA breaks and ATM/DNA-PKcs signaling to nuclear factor kappa B (NF-kB) and IRF activation. Since the majority of the work has been done in epithelial cells or mouse embryonic fibroblasts, the cell-autonomous role of the DDR in instructing pro-inflammatory macrophage programs is less well understood. Nonetheless, irradiation, beyond inducing ATM-IRF1-dependent interferon responsive genes (as above), also upregulated IRF5 via ROS-mediated ATM activation in the murine RAW264.7 macrophage cell line [47] (**Figure 3B**). IFN-γ stimulation of human monocyte-derived macrophages and murine RAW264.7 cells similarly induced ATM-dependent IRF5 expression (**Figure 3B**). These studies did not address whether elevated ROS was the cause or consequence (or both) of irradiation-induced DDR activation.

## RECIPROCAL REGULATION OF THE DDR BY TYPE I IFNs IN BACTERIAL INFECTION

We already discussed that ATM suppresses type I IFN responses at steady state, following MCSF stimulation [40], and after bacterial or viral infection [43]. Type I IFNs reciprocally promote DDR activation, establishing a positive feedback loop [48]. Bacterial infection of BMDM with *L. monocytogenes* leads to dsDNA breaks, which activates ATM and DNA-PKcs (**Figure 3C**). Type I IFNs were required for this activation, with *Ifnar1*^-/-^ macrophages showing undetectable levels of γH2AX after infection. Thus, tonic ATM activation at homeostasis suppresses MCSF-induced type I IFNs [40] and type I IFNs induced by bacterial infection are required for DDR activation via ATM and DNA-PKcs [48] (**Figure 3C**). Whether type I IFNs may similarly regulate basal ATM activation by MCSF at homeostasis remains unclear.

## DDR-MEDIATED INFLAMMASOME ACTIVATION FOLLOWING BACTERIAL INFECTION OR LPS/IFN-γ

Does bacterial and IFN-induced DDR activation re-program macrophages? This held true for BMDM from mice with a combined severe combined immunodeficiency (SCID, harboring a non-functional DNA-PKcs protein, [49]) and conditional *Atm* deficiency background [50], while BMDM from mice with either SCID or *Atm* deficiency alone did not show an activated DDR after infection and had subtler differences in their genetic programs compared to wild type mice. *Scid; Atm*^*fl/fl*^*- Lyz2*-Cre BMDM remarkably expressed significantly reduced IL-1β following bacterial infection with *L. monocytogenes*, while BMDM macrophages from SCID mice further showed decreased IL-18 production following infection [48].

Beyond *L. monocytogenes* infection, BMDM stimulation with a combination of LPS and IFN-γ but not LPS or IFN-γ alone, led to dsDNA break induction and DDR activation via combined activation of ATM and DNA-PKcs in BMDM [48]. In addition, *Atm* deficient BMDM infected with *Streptococcus pneumoniae* showed decreased IL-1β and decreased inflammasome activation [51]. Overall, these studies suggest that significant macrophage activation by LPS and IFN-γ and/or bacterial infection induces genotoxic stress that re-programs activated macrophages promoting inflammasome activation via IFN-mediated activation of the DDR.

## BACTERIA INDUCED GENOTOXIC STRESS VIA NO AND ATM PROMOTES INFLAMMASOME ACTIVATION BY SUPPRESSING ROS

How do bacterial stimuli induce dsDNA breaks? Two prime suspects in this process have been ROS and NO. LPS is known to induce ROS and NO production by inducing metabolic shifts in glycolysis and the accumulation of succinate and citrate in the tricarboxylic acid cycle (TCA) [52]. How these metabolic processes influence the DDR and its downstream processes is a question in need of further exploration. *Atm* deficient BMDM expressed increased amounts of ROS and displayed reduced inflammasome activation following stimulation with LPS or infection with *Salmonella typhimurium* or *S. pneumoniae* [51] (**Figure 3C**). ROS inhibition restored inflammasome activation suggesting that ROS negatively regulates bacterial-induced inflammasome activation in *Atm* deficient hosts. Whether ROS also cause dsDNA breaks, thus amplifying DDR activation following bacterial infection remained unclear.

BMDM from mice deficient in *Nos2* (encoding the Nitric Oxide Synthase, iNOS), but not BMDM from mice deficient in *Cybb* (encoding gp91^phox^, a subunit of the membrane-bound NADPH oxidase that catalyzes the formation of superoxide (O_2_^−^), precursor of hydrogen peroxide (H_2_O_2_) and other antimicrobial oxidants [53]), had reduced γH2AX levels following stimulation with LPS and IFN-γ suggesting that NO [48], rather than ROS, mediates DNA damage and thus activates the DDR. In agreement, prior work on a macrophage cell line showed that LPS induced dsDNA breaks via peroxynitrite (produced by the reaction of NO with superoxide anion) [54]. However, BMDM stimulated with LPS or LPS and IFN-γ had increased levels of γH2AX earlier than the time of NO production, and coinciding with increased levels of ROS. Furthermore, mitochondrial ROS scavengers suppressed γH2AX levels, while macrophages stimulated with LPS and IFN-γ showed increased levels of 8-hydroxyguanosine (a marker of oxidative stress), corroborating that ROS mediates dsDNA breaks. It is possible though that the role of ROS vs NO and/or peroxynitrite changes over time, with ROS mediating oxidative stress early, while NO and/or peroxynitrite may potentially contribute to the activation of the DDR following chronic stimulation with inflammatory ligands (**Figure 3C**). Indeed, treating BMDM with an NO donor induced increased γH2AX levels [55]. It is important to note that only two of the above studies have demonstrated dsDNA breaks with a comet assay [48, 54], raising the possibility that mitochondrial ROS-mediated genotoxic stress may be induced by other forms of DNA damage. In particular, whether stalled replication forks, immunostimulatory ssDNA in the cytoplasm [43], or mitotic defects leading to the formation of micronuclei [56] and potentially thus activating cyclic GMP-AMP synthase (cGAS)-STING signaling [57], regulate the DDR activation following bacterial infection, remains unclear.

## THE DDR AT THE CROSSROADS OF GENOTOXIC SIGNALING, ANTIVIRAL PROGRAMS AND AUTOIMMUNITY

Given the role of the DDR in regulating type I IFNs in the context of homeostatic macrophage responses to MCSF and antibacterial defense, it is tempting to place cell-autonomous DDR signaling in innate immune cells at the center of antiviral, autoinflammatory and autoimmune responses. A continuously growing body of literature illustrates how activation of the DDR in epithelial cells or fibroblasts primes innate immunity [58–60]. The pathways however that link cell-autonomous DDR activation in innate cells to their cell type-specific programs are far less understood.

## NUCLEIC ACID METABOLIC PATHWAYS CONTROL ANTIVIRAL PROGRAMS AND AUTOINFLAMMATION

**Figure 4:**
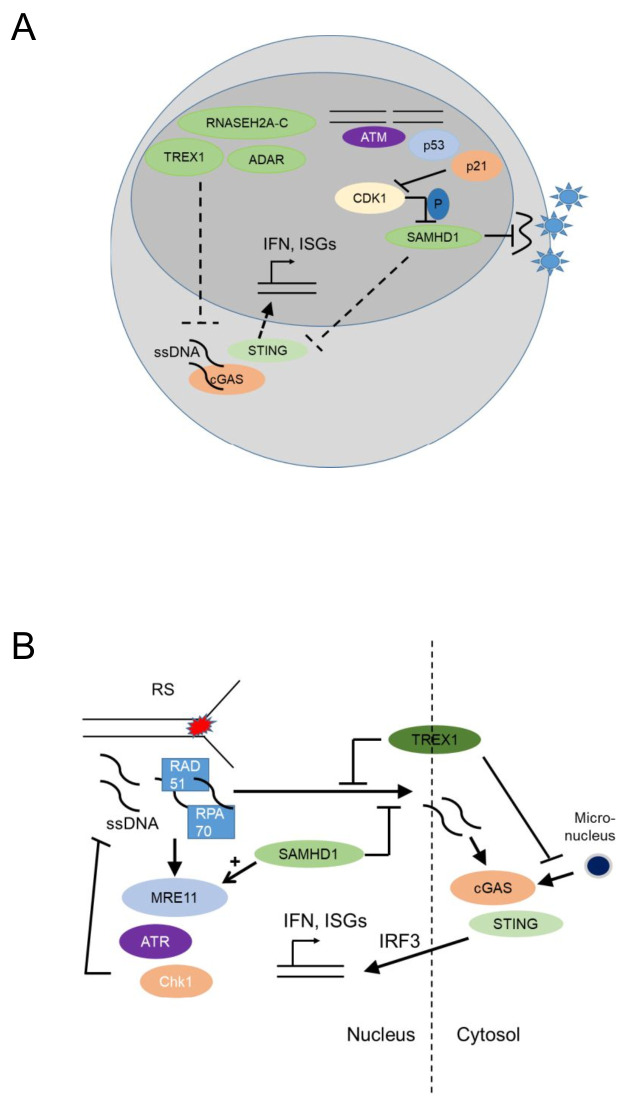
FIGURE 4: Nucleic acid metabolic pathways control antiviral programs and autoinflammation and may have genome-protecting functions by suppressing cytoplasmic cGAS activation and type I interferon responses. **(A)** Nucleic acid-metabolizing enzymes SAMHD1 (SAM domain and HD domain-containing protein 1), TREX1 (three prime repair exonuclease 1), RNASEH2A-C (Ribonuclease H2, Subunit A, C-Term), and ADAR (Adenosine Deaminase RNA Specific), control systemic type I interferon (IFN) levels by negatively regulating the cGAS-STING pathway. Unphosphorylated SAMHD1 restricts viral replication in myeloid cells either by depleting the dNTP pool or through its RNAse activity. This antiviral activity of SAMHD1 is suppressed upon SAMHD1 phosphorylation by cyclin A2/Cdk1 in cycling cells, while genotoxic stress that leads to p53 and p21 upregulation and subsequently CDK1/2 (cyclin-dependent kinase 1/2) suppression and SAMHD1 dephosphorylation increase this antiviral activity. **(B)** SAMHD1 promotes degradation of ssDNA at stalled replication forks by stimulating the exonuclease activity of MRE11 and thus activating ATR-CHK1 to alleviate replication stress. This prevents ssDNA accumulation in the cytosol where they would activate cGAS-STING leading to increased expression of type I interferons. TREX1 (three prime repair exonuclease 1) also promotes the removal of ssDNA molecules (formed due to replication stress) through RPA70 (Replication protein A 70 kDa DNA-binding subunit) and Rad51, thus preventing leakage of ssDNA to the cytosol and phosphorylation of IRF3. TREX1 also limits cGAS activation at micronuclei, by degrading micronuclear DNA. Replication stress, ssDNA and micronuclei promote the cGAS – STING – type I IFN activation pathway.

The myeloid restriction factor sterile alpha motif domain and HD domain-containing protein 1 (SAMHD1) is a deoxynucleoside triphosphate (dNTP) triphosphohydrolase and a ribonuclease that is well known as a restriction factor for HIV replication in myeloid cells [61, 62]. Interestingly, in its unphosphorylated state, SAMHD1 restricts HIV replication in a cell-type specific manner, namely in dendritic and myeloid cells. This antiviral activity of SAHMD1 is suppressed upon phosphorylation by cyclin A2/cyclin dependent kinase 1(Cdk1) in cycling cells [63] (**Figure 4A**). The virus counteracts this restriction by expressing Vpx, a protein that induces proteasomal degradation of SAMHD1 [64]. Initially it was thought that SAMDH1 blocks reverse transcription of retroviral RNA into DNA by depleting cellular dNTPs [65]. However, phosphorylation of SAMHD1 by cyclin A2/Cdk1 in cycling cells suppresses its ability to restrict HIV1 [63] but it does not regulate intracellular dNTP levels, though this point is a matter of dispute [66]. This conundrum might be explained by the finding that SAMHD1 possesses RNAse activity and the RNase but not the dNTPase function is essential for HIV restriction [67].

SAMHD1 has an antiviral activity in nondividing macrophages not only for retroviruses but also for DNA viruses. Restriction of herpes simplex virus 1 HSV1 replication required the dNTP triphosphohydrolase activity of SAMHD1 and was partially overcome by addition of exogenous dNTPs. While SAMHD1 phosphorylation was important for its antiretroviral activity, restriction of HSV1 was not affected by SAMHD1 phosphorylation status [68].

Loss-of function mutations in the nucleic acid-metabolizing genes *SAMHD1*, three prime repair exonuclease 1 (*TREX1)*, ribonuclease H2 subunit A-C (*RNASEH2A-C)*, and Adenosine Deaminase RNA Specific *ADAR*, result in elevated systemic type I IFN levels due to activation of the cGAS-STING pathway [69–71] and cause Aicardi-Goutieres Syndrome (AGS), an infancy-onset inflammatory encephalopathy that phenotypically mimics congenital viral infection and shows overlap with systemic lupus erythematosus (SLE), a prototypic autoimmune disease characterized by autoantibodies against nucleic acids (**Figure 3B**) [72]. AGS is part of a broader group of genetic diseases characterized by chronically elevated type I IFNs, systemic autoinflammation and autoimmunity and called ‘interferonopathies' (reviewed in [73–75]).

The contribution of innate cell-intrinsic deficiency of nucleic acid metabolizing enzymes to systemic interferonopathies is of high interest. Unconditional *Trex1* deficiency in mice leads to an autoinflammatory disease with immune infiltrates in skeletal and heart muscle, gastric mucosa [76]. Conditional Trex1 deficiency in CD11c or CX3CR1 expressing myeloid cells was sufficient to induce systemic inflammation, in contrast to Trex1 deficiency targeted to keratinocytes and fibroblasts [77]. Conditional Trex1 deficiency driven by the dendritic cell (DC)-specific Clec9a Cre also induced systemic autoinflammatory disease, although less severe than the one induced by hematopoietic Tie2- or myeloid CD11c or CX3CR1-Cre expression [77]. It is possible that *Trex1* deficiency triggers myeloid-specific pathways that may initiate systemic inflammation, whereas Trex1 deficiency in epithelial cells may act to further amplify auto-inflammatory responses, however this hypothesis remains to be experimentally proven.

## NUCLEIC ACID METABOLIZING ENZYMES HAVE GENOME PROTECTIVE FUNCTIONS

In malignant epithelial cells, SAMDH1 was found to promote DNA End Resection to facilitate DNA repair by homologous recombination [78], while SAMHD1 depletion in fibroblasts promoted accumulation of R-loops at transcription-replication conflict regions, and activated the DDR [79]. Thus, SAMHD1 may have canonical genome-protecting functions, as well as myeloid cell-specific antiviral activity. In agreement with this notion, SAMHD1 promoted degradation of ssDNA at stalled replication forks in human epithelial and immortalized B cell lines [80] by stimulating the exonuclease activity of MRE11 and thus activating ATR-CHK1 to alleviate replication stress. SAMHD1 deficiency resulted in ssDNA accumulation in the cytosol where they activated cGAS-STING and induced type I IFNs [80] (**Figure 4B**). Thus, SAMHD1 promotes DNA repair and prevents replication stress, establishing that nucleic acid metabolism promotes genomic stability.

TREX1, the major 3' DNA exonuclease in mammalian cells, also functions to maintain genomic stability since *Trex1* deficient fibroblasts showed chronic ATM-dependent p53 activation [81]. Interestingly, this was accompanied by defective G1/S transition and accumulation of ssDNA molecules, produced in the S phase, in the ER [81], suggesting that TREX1 functions to remove ssDNA molecules formed due to replication stress (**Figure 4B**). RPA70 and Rad51, both well-known components of the replication stress response, bound ssDNA microinjected in the nucleus of epithelial cells and prevented leakage of ssDNA to the cytosol and phosphorylation of IRF3 [82]. Trex1 deficient fibroblasts showed increased binding of RPA and Rad51 to ssDNA, increased ssDNA breaks, and increased IRF3 phopshorylation, establishing that TREX1 prevents replication stress and suppresses a replication stress-dependent type I IFN response (**Figure 4B**). Its localization with a tail anchored at the outer nuclear membrane and the C-Terminus oriented into the ER lumen [82] suggested that TREX1 may function to prevent the accumulation of ssDNA leaking into the cytoplasm of cells undergoing replication stress. More recently, TREX1 was additionally found to limit cGAS activation at micronuclei (**Figure 4B**), formed due to chromosome missegregation following replication stress, by degrading micronuclear DNA upon micronuclear envelope rupture [83].

How accumulation of ssDNA in the cytoplasm promotes replication stress in TREX1 deficient cells remains unclear. It is possible that TREX1 prevents replication stress by translocating to the nucleus in the S-phase [81], while its perinuclear location is additionally important for the suppression of type I IFN responses induced by ssDNA leaking to the cytoplasm of cells undergoing replication stress. Overall, both SAMHD1 and TREX1 protect from replication stress and suppress type I IFN activation by suppressing cGAS-STING activation.

## NUCLEAR cGAS PROMOTES GENOMIC INSTABILITY

In contrast to the genome-protective functions of nucleic acid metabolizing enzymes, activation of nuclear cGAS may be a pathway promoting genomic instability [84, 85]. cGAS is well known as a cytoplasmic DNA sensor that activates the STING-IRF3-type I IFN signaling pathway [86]. Two recent studies have revealed that nuclear cGAS suppresses DNA repair via HR and thus may promote tumorigenesis [85, 87]. In summary, nucleic acid metabolizing enzymes promote genomic stability and their deficiency leads to replication stress and cGAS-STING-mediated activation of type I iIFN responses (**Figure 4B**). The role of nuclear cGAS-mediated genomic instability, as potential amplifier of auto-inflammation in individuals deficient in nucleic acid metabolizing enzymes, remains unclear.

## TLR SIGNALING AND GENOTOXIC STRESS REGULATE NUCLEIC ACID METABOLISM IN MACROPHAGES

Intriguingly, SAMHD1 de-phosphorylation is part of the macrophage response to inflammatory stimuli. Thus, viruses, but also the TLR4 ligand LPS induce p21 upregulation, CDK1 depletion, G0 arrest and SAMHD1 de-phosphorylation, thus enhancing the transcriptional activity of NF-kB and IRF7 [88, 89] and the upregulation of type I IFNs in macrophages, while IL12/IL18 signaling in primary macrophages increased expression of SAMHD1 and SAMHD1-dependent HIV1 restriction [90] providing a link between inflammatory stimuli, cell cycle control and macrophage function, a link that may be required for macrophage genomic stability.

SAMHD1 is further directly regulated by genotoxic stress [91, 92]. SAMHD1 expression is induced by topoisomerase inhibitors [92] and restricts HIV replication in human monocyte-derived macrophages. The Etoposide-induced antiviral effect was associated with activation of p53, p21, increased expression of CDK1 and de-phosphorylation of SAMHD1. Etoposide-induced antiviral activity in macrophages was completely reversed by SAMHD1 depletion [92]. Overall, genotoxic stress-induced and DDR-mediated cell cycle arrest promote de-phosphorylation of SAMHD1, which promotes antiviral programs.

Genotoxic stress induced by γ-irradiation (inducing dsDNA breaks) or treatment with hydroxyurea (inducing replication stress) may further regulate TREX1 by inducing its re-localization from the endoplasmic reticulum to the nucleus in the S phase [81]. Thus, DDR signaling is embedded in pathways that regulate nucleic acid metabolism and are regulated by classical genotoxic stress-inducing treatments or inflammatory stimuli. The role of such pathways in shaping the programs of proliferating myeloid precursors is not well understood.

## THE DDR MAY CONTROL MULTINUCLEATED MACROPHAGE FITNESS

Cellular fitness confers a proliferation and/or survival advantage. In the case of cancer cells this may determine their resistance or susceptibility to chemotherapeutic agents. Signaling pathways conferring cellular fitness may be particularly important for osteoclasts, a multinucleated, bone-resorbing cell of the macrophage lineage, essential for bone homeostasis, as well as multinucleated macrophages that are histologic hallmarks of granulomas, organized immune aggregates arising during persistent inflammatory stimuli. Both of these cell types acquire and maintain a large cellular size and a long lifespan [93, 94], suggesting demanding metabolic requirements. There is very little known on the role of the DNA damage response in conferring osteoclast and granuloma macrophage fitness; however, two studies have shown a differential contribution of ATM and ATR in this process [56, 95].

**Figure 5:**
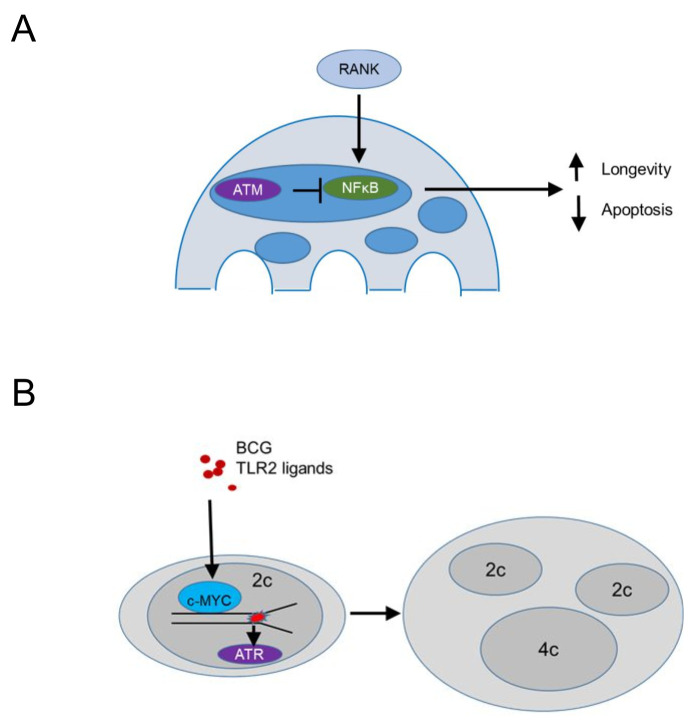
FIGURE 5: The DNA damage response may control multinucleated macrophage cell fitness. **(A)** In osteoclasts RANK (Receptor Activator of NF-κB) signaling activates the NFκB (nuclear factor 'kappa-light-chain-enhancer' of activated B-cells) pathway promoting survival, an effect inhibited by ATM, leading to increased apoptosis. **(B)** Chronic bacterial infection and TLR2 ligands upregulate c-MYC and induce replication stress, which causes cytokinesis failure. ATR promotes growth, survival and genomic stability of polyploid macrophages in granulomatous microenvironments.

Conditional deletion of *Atm* driven by a Cathepsin K Cre (specific for osteoclasts and multinucleated macrophages in granulomas) demonstrated that ATM promotes apoptosis and limits the lifespan of steady state osteoclasts [95] (**Figure 5A**). The population size of *Atm*-deficient osteoclasts was reduced *in vivo*, while *ex vivo* MBDM precursors treated with recombinant RANKL and MCSF showed no defect in macrophage formation. TUNEL staining revealed significantly reduced apoptotic nuclei in tibial osteoclasts of *Atm*-deficient, 3-week old mice, a time point where osteoclast precursors proliferate and BrdU+ nuclei are identified in 30% of osteoclast nuclei [96] compared to the wild type controls, suggesting that ATM promotes apoptotic cell death (**Figure 5A**). *In vitro*, osteoclasts lacking ATM survived longer and expressed reduced levels of cleaved Caspase 3 after RANKL/MCSF withdrawal [95]. Osteoclasts lacking ATM showed *in vitro* increased levels p-p65 suggesting that survival was mediated by enhanced NF-kB signaling (**Figure 5A**). This is in agreement with the role of RANKL in progestin-driven mammary cancer epithelial cells [97], where RANK signaling protects cancer cells from irradiation-induced and DDR-mediated cell death. Overall, these data suggest that ATM suppresses RANK-mediated and NF-kB dependent survival in steady state osteoclasts or their precursors, while the mechanism by which ATM is activated in osteoclasts or their precursors remains unclear.

In contrast, persistent stimulation of BMDM precursors *in vitro* with bacterial lipoproteins binding TLR2 or infection with *Mycobacterium bovis* Bacillus Calmette-Guérin (BCG) induced macrophage precursor replication stress, demonstrated by a DNA fiber assay [56] (**Figure 5B**). The presence of growth factors, including MCSF, promoted macrophage polyploidy via c-Myc, since pharmacologic inhibition of c-Myc suppressed cell cycle progression and replication stress. The data supported prior work in cancer cell lines suggesting that growth factor signaling may be a pathway to overcome p53-mediated restrictions to the proliferation of tetraploids [98]. In agreement, BMDM treated with TLR2 ligands showed increased p53 expression [55] and p53 deficient BMDM formed significantly more multinucleated cells following treatment with TLR2 ligands. These data suggest that bacterial lipoproteins induce replication stress and activate p53 that acts to limit multinucleation and polyploidy. In contrast, growth factor signaling via c-Myc promotes a bypass of p53-mediated restrictions in macrophage ploidy. This process was additionally promoted by ATR, since pharmacologic inhibition of ATR inhibition promoted chromosome missegregation and suppressed macrophage polyploidy, suggesting that ATR signaling safeguards the genomic stability of multinucleated macrophages (**Figure 5B**). *In vivo* granuloma macrophages in mice and humans showed increased levels of γH2AX and mixing CD45.1 with CD45.2 bone marrow chimeras provided evidence that granuloma macrophages undergo multinucleation due to mitotic defects. These studies do not exclude the possibility that multinucleated giant cells, a well-known hallmark of granulomas, form by cell-to-cell fusion (rather than replication stress-induced mitotic defects). Rather, they give rise to the notion that replication stress leading to multinucleation is a hallmark of growth-factor-dependent macrophage fitness in chronic inflammatory responses. Thus, large fusion-derived multinucleated giant cells, mitotic-defect-derived polyploid macrophages, and epithelioid macrophages co-exist within one inflammatory microenvironment that provides fitness-promoting growth factors, thus supporting granuloma growth.

## CONCLUDING REMARKS

Homeostatic and inflammatory challenges impose genotoxic stress in macrophages and their precursors, leading to activation of the DDR. Apart from its canonical functions in safeguarding genome stability, the DDR can also activate cell type specific transcriptional programs that are largely unexplored. Bacterial infection induces genotoxic stress in multiple forms including dsDNA breaks and oxidative stress. ATM-mediated signaling plays a crucial role in suppressing inflammatory responses. Viral infection and defects in nucleic acid metabolizing enzymes are linked to replication stress, leading to enhanced type I IFN responses. The role of the DDR in innate cells in this context remains unclear and it is tempting to hypothesize that ATR acts to limit systemic levels of type I IFN and autoimmunity. Finally, how genotoxic stress shapes the fitness of long-lived and large sized macrophage populations is only now being explored. Dissecting the pathways that integrate cell-autonomous genotoxic signaling in the diversity of genetic programs adopted by macrophages to maintain homeostasis and drive inflammation will be an important avenue for future research.

## Abbreviatons:

AGS: – Aicardi-Goutieres Syndrome;
ATM: – Ataxia telangiectasia mutated;
ATR: – Ataxia telangiectasia and Rad3-related;
BMDM: – bone marrow-derived macrophage;
cGAS: – cyclic GMP-AMP synthase;
DDR: – DNA Damage Response;
DNA-PK: – DNA-dependent protein kinase;
DNA-PKcs: – DNA-PK catalyitc subunit;
dNTP: – deoxynucleoside triphosphate;
ds: – double strand;
HR: – homologous recombination;
IFN: – interferon;
IL: – interleukin;
IRF: – IFN regulatory factor;
LPS: – lipopolysaccharide;
MCMV: – murine cytomegalovirus;
MCSF: – macrophage colony stimulating factor;
MRN: – Mre11-Rad50-NBS1;
NHEJ: – non-homologous end joining;
NK: – natural killer;
NO: – nitric oxide;
RAG: – recombination-activated gene;
ROS: – reactive oxygen species;
ss: – single strand;
SCID: – severe combined immunodeficiency;
STING: – stimulator of interferon genes;
TLR: – toll-like receptor.
